# Long non-coding RNA MIAT promotes breast cancer progression and functions as ceRNA to regulate DUSP7 expression by sponging miR-155-5p

**DOI:** 10.18632/oncotarget.19190

**Published:** 2017-07-12

**Authors:** Tian Luan, Ximei Zhang, Shuyuan Wang, Yan Song, Shunheng Zhou, Jing Lin, Weiwei An, Weiguang Yuan, Yue Yang, Huilong Cai, Qingyuan Zhang, Lihong Wang

**Affiliations:** ^1^ Institute of Cancer Prevention and Treatment, Heilongjiang Academy of Medical Science, Harbin Medical University, Harbin 150081, China; ^2^ Department of Histology and Embryology, Harbin Medical University, Harbin 150081, China; ^3^ College of Bioinformatics Science and Technology, Harbin Medical University, Harbin 150081, China; ^4^ Department of Internal Medicine, The Third Affiliated Hospital of Harbin Medical University, Harbin 150081, China; ^5^ Department of Pathophysiology, School of Medicine, Southeast University, Nanjing 210009, China

**Keywords:** MIAT, breast cancer, ceRNA, miR-155-5p, DUSP7

## Abstract

Long non-coding RNAs (lncRNA) have been reported as key regulators in the progression and metastasis of breast cancer. In this study, we found that the lncRNA myocardial infarction associated transcript (MIAT) expression was upregulated in breast cancer in The Cancer Genome Atlas (TCGA) data sets. We validated that MIAT was higher in breast cancer cell lines and advanced breast tumors than in normal controls. And MIAT overexpression associated with TNM stage and lymphnode metastasis. Knockdown MIAT inhibited breast cancer cell proliferation and promoted apoptosis. Also MIAT downregulation suppressed epithelial-mesenchymal transition (EMT) and decreased migration and invasion in MDA-MB-231 and MCF-7 breast cancer cell lines. More importantly, knockdown MIAT inhibited tumor growth *in vivo*. Our results suggested that MIAT acted as a competing endogenous RNA (ceRNA) to regulate the expression of dual specificity phosphatase 7 (DUSP7) by taking up miR-155-5p in breast cancer. There were positive correlation between MIAT and DUSP7 expression in breast cancer patients. We conclude that MIAT promotes breast cancer progression and functions as ceRNA to regulate DUSP7 expression by sponging miR-155-5p in breast cancer.

## INTRODUCTION

Breast cancer is the most common malignancy in women and the incidence increases gradually all over the world [1, 2]. Thus, a detailed understanding of the basis for breast cancer is important. Long noncoding RNAs (lncRNAs) are long RNA molecules (>200nt) that regulate gene expression at transcriptional, post-transcriptional and translational regulation levels [3]. Emerging evidence has suggested lncRNA as a new class of players involved in the development and progression of cancer [4, 5]. However, the regulatory roles played by lncRNAs in breast cancer are largely unknown.

LncRNA myocardial infarction associated transcript (MIAT), mapped to human chromosome 12q12.1 with 5 exons, was originally reported to be associated with a susceptibility to myocardial infarction [6], also abundantly expressed in nervous system [7] and retinal tissue [8]. Recently some studies suggested that MIAT was selectively upregulated in neuroendocrine prostate cancer [9] and in aggressive form of chronic lymphocytic leukemias [10]. Due to the pan-cancer analysis project [11, 12], we investigated whether MIAT could be potential breast cancer diagnostic biomarker. Using bioinformatics methods, we also found that MIAT expression was higher in breast cancer tissues than in normal breast tissues from The Cancer Genome Atlas (TCGA) breast cancer data sets. Then we studied the molecular mechanism of MIAT in breast cancer. Since Pandolfi PP. and his groups presented a competing endogenous RNA (ceRNA) hypothesis in 2011 [13], increasing evidence has been provided that lncRNAs as ceRNAs were involved in cancer onset and progression [5, 14]. It has been reported that MIAT regulated microvascular dysfunction through acting as a ceRNA by sponging miR-150-5p [15, 16]. We analyzed whether MIAT could function as ceRNA in breast cancer. A bioinformatics analysis showed that there were conserved binding sites for both MIAT and the 3′-UTR of DUSP7 mRNA on miR-155-5p. DUSP7 belongs to a class of DUSPs, is a cytosolic MKP that dephosphorylates the extracellular signal-regulated kinases (ERK1/2), c-Jun N-terminal kinases (JNK) and p38 kinase [17, 18]. And DUSP7 has been shown to be overexpressed in peripheral blood mononuclear cells and bone marrow from patients diagnosed with acute leukemia [19, 20].

In this study, we found MIAT was upregulated in breast cancer in TCGA data sets. We also validated that the expression of MIAT was higher in breast cancer cell lines and advanced breast tumors than in normal controls. Knockdown MIAT led to inhibit cell proliferation, migration, invasion and epithelial-mesenchymal transition (EMT) while promote apoptosis in breast cancer cells. Moreover, downregulation of MIAT inhibited the tumor growth *in vivo*. To further extend this finding, we investigated that MIAT acted as a ceRNA to regulate DUSP7 mRNA by taking up miR-155-5p in breast cancer cells, which might be one of the mechanisms of MIAT functions as an oncogene.

## RESULTS

### MIAT expression was upregulated in breast cancer cell lines and tissues

**Figure 1:**
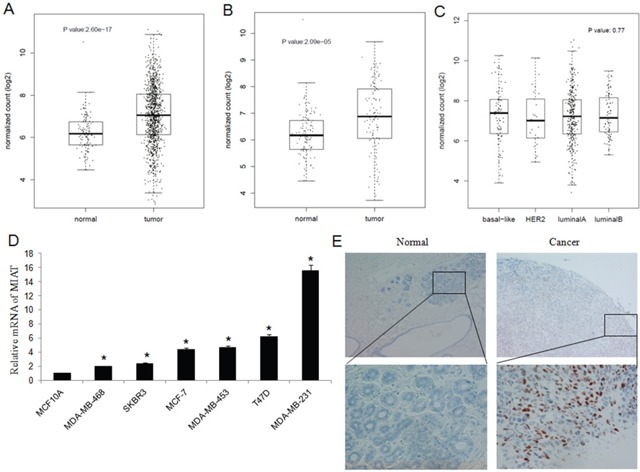
Expression of MIAT in TCGA, breast cancer cell lines and tissues **(A)** MIAT expression was higher in breast cancer tissues than in normal breast tissues from TCGA dataset (*P*<0.05). **(B)** MIAT expression was higher in breast cancer tissues than in paired normal breast tissues from TCGA dataset (*P*<0.05). **(C)** There were no significance of MIAT expression among different breast cancer subtypes from TCGA dataset (*P*=0.77). **(D)** MIAT expression was higher in breast cancer cell lines than in normal breast epithelial cell line MCF-10A. ^*^P <0.05, Two-side Student's t-test; n = 3. **(E)** RNA Scope^®^ technology detection showed that MIAT RNA was significant overexpression in human breast cancer tissues than in adjacent normal tissues (up ×100; bottom ×400).

We analyzed the expression of MIAT in TCGA breast cancer data sets using bioinformatics methods. The results revealed that MIAT was upregulated in breast cancer tissues than in normal breast tissues (*P*<0.05) (Figure 1A) and in paired case-control analysis MIAT expression was still upregulated in breast cancer tissues (*P*<0.05) (Figure 1B). When we classified the breast cancer patients according to molecular subtypes, we found there were no difference of MIAT level among breast cancer subtypes (*P*=0.77) (Figure 1C).

To validated the results from TCGA, we detected MIAT mRNA level in the human normal breast epithelial cell line MCF-10A and breast cancer cell lines MCF-7, MDA-MB-231, MDA-MB-468, T-47D, SK-BR-3 and MDA-MB-453. We found that MIAT expression was higher in cancer cell lines than in MCF-10A (Figure 1D).

Next, we investigated the expression of MIAT in human breast tumor tissue microarrays (TMA). We detected MIAT mRNA in 30 paired breast cancer tissues by RNA Scope^®^ 2.0 technology and found that MIAT mRNA was higher in breast cancer tissues than in normal breast tissues (Figure 1E) (Supplementary Table 1) (*P*<0.05). The association between MIAT expression and clinicopathologic parameters was also tested by the chi-square tests. The results suggested that MIAT expression was higher in breast cancer patients with TNM III stage (*P*<0.01) (Supplementary Table 2). And further we found that 11 patients with MIAT positive were all lymphnode metastasis and 12 patients without lymphnode metastasis were MIAT negative (*P*<0.01) (Supplementary Table 3), which indicated that MIAT expression was positively associated with lymphnode metastasis. Our above results suggested that MIAT upregulation may be associated with the occurrence and metastasis of breast cancer.

### MIAT knockdown inhibited breast cancer cell proliferation and promoted apoptosis

**Figure 2:**
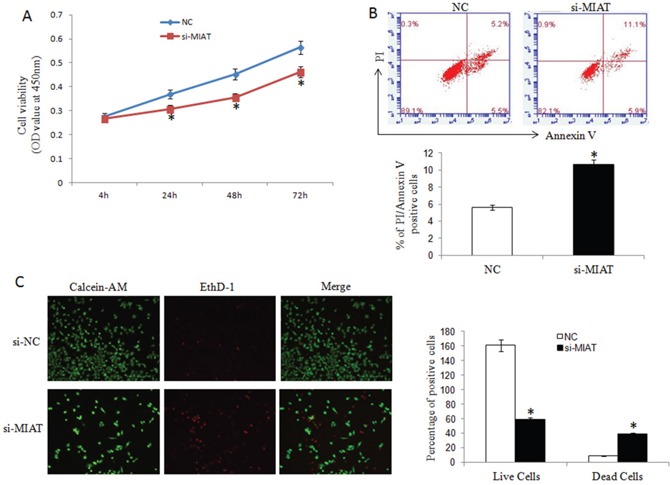
MIAT downregulation inhibited breast cancer cell proliferation and promoted apoptosis **(A)** CCK-8 assays were performed to measure the proliferation of MDA-MB-231 cells. *P< 0.05, Two-side Student's t-test; n=3. **(B)** MIAT downregulation promoted apoptosis as shown by increased PI and Annexin V positive cells. ^*^P <0.05, Two-side Student's t-test; n = 3. **(C)** Cell apoptosis was analyzed using Calcein-AM/EthD-1 staining. Green: live cells, Red: dead or dying cells (×100). ^*^P <0.05, Two-side Student's t-test; n = 3.

To investigate the function of MIAT in breast cancer, we transfected MIAT-siRNA or scramble siRNA into MDA-MB-231 cells to knockdown MIAT. We detected the cell proliferation by CCK8 at 4h, 24h, 48h and 72h time point and found that interfered MIAT expression inhibited cell proliferation (Figure 2A). And using flow cytometry, we analyzed the apoptosis rate of MDA-MB-231 cells transfected with MIAT-siRNA or scramble siRNA. The data suggested that MIAT downregulation promoted apoptosis as shown by increased PI and Annexin V double positive cells (Figure 2B). And also EthD-1/Calcein-AM staining revealed that MIAT siRNA but not scrambled siRNA transfection further increased the number of dead or dying cells (Figure 2C).

### MIAT downregulation inhibited breast cancer cell migration, invasion and EMT

**Figure 3:**
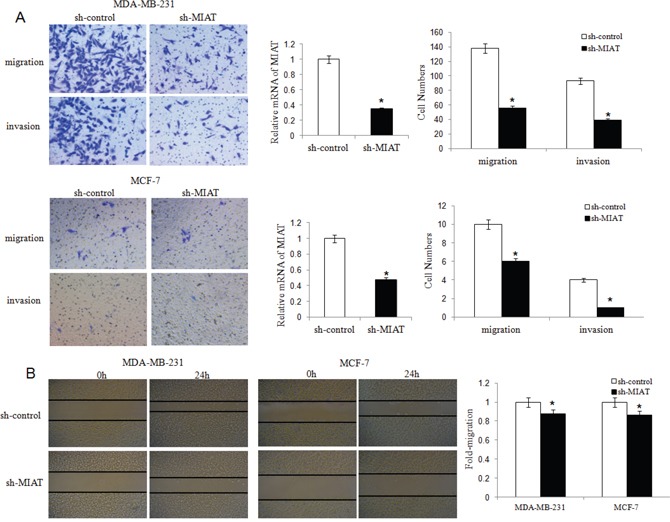
MIAT downregulation inhibited migration and invasion of MDA-MB-231 and MCF-7 breast cancer cells **(A)** Transwell migration and invasion assays were used to evaluate MDA-MB-231 and MCF-7 cell numbers with or without MIAT downregulation. Cells were stained with crystal violet after cultured 24h and counted for statistical analysis (×200). The qRT-PCR was done to verify the MIAT expression level. ^*^P <0.05, Two-side Student's t-test; n = 3. **(B)** The scratch-wound gap of MDA-MB-231 and MCF-7 cells with or without MIAT downregulation was photographed before and after 24 h incubation. After performing the wound healing test, the results were analyzed by measuring the range of migrating cells from 3 different fields for each wound (×50). ^*^P <0.05, Two-side Student's t-test.

**Figure 4:**
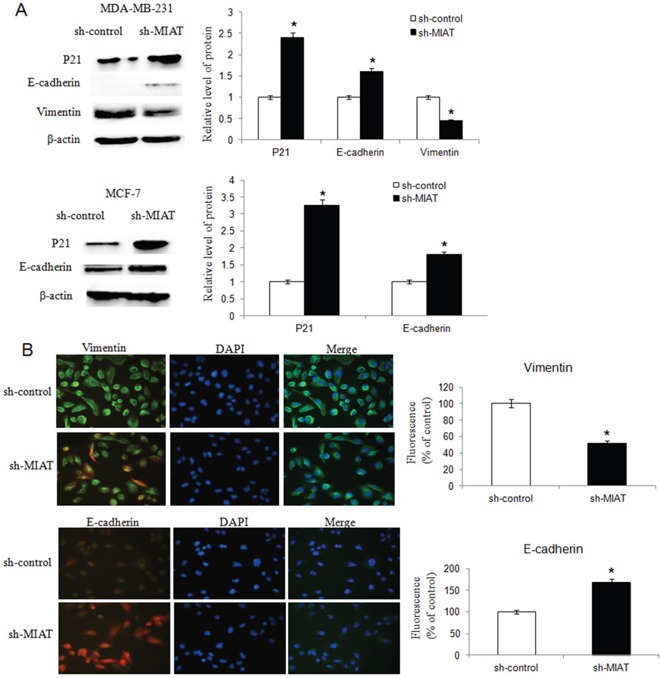
MIAT downregulation inhibited breast cancer cell EMT **(A)** Western blot analysis showed that knockdown of MIAT increased P21 and E-cadherin expression both in MDA-MB-231 and MCF-7 cells. The vimentin level was inhibited in MDA-MB-231 cell after MIAT knockdown but no vimentin was found in MCF-7 cell (data not shown). β-actin was detected as the loading control. ^*^P <0.05, Two-side Student's t-test; n = 3. **(B)** Immunofluoresence assay was conducted to detect vimentin and E-cadherin expression in MDA-MB-231 cells. The expression of vimentin was lower but E-cadherin was higher in cells transfected with sh-MIAT than in sh-control cells. Nuclei, blue; vimentin (up) or E-cadherin (bottom), green; Cells transfected with lentivirus, red (×400). Results were presented as a relative percentage to control (defined as 100%). ^*^P <0.05, Two-side Student's t-test; n = 3.

We established MDA-MB-231 and MCF-7 cell lines with stably downregulation MIAT expression by lentivirus vector. We found that MIAT downregulation decreased both MDA-MB-231 and MCF-7 cells migration and invasion ability (Figure 3A and 3B). Furthermore, we detected the expression of P21 to analyzed if MIAT involved in cell cycle. The results showed that P21 level was higher in sh-MIAT cells than in sh-control cells (Figure 4A), which indicated MIAT knockdown promoted cell cycle arrest. Then we analyzed vimentin and E-cadherin expression by western blot to investigate whether MIAT induced breast cancer cell EMT. As shown in Figure 4A, E-cadherin expression level was increased both in sh-MIAT MDA-MB-231 and MCF-7 cells compared with sh-control cells. While vimentin expression level was decreased in sh-MIAT MDA-MB-231 and no expression in MCF-7 cell (data not shown). We further examined the expression of vimentin and E-cadherin in MDA-MB-231 cell by immunofluorescence staining and found the same results with western blot (Figure 4B). The data suggested that MIAT knockdown could inhibit EMT of breast cancer cells.

### Knockdown of MIAT inhibited breast cancer cell growth *in vivo*

**Figure 5:**
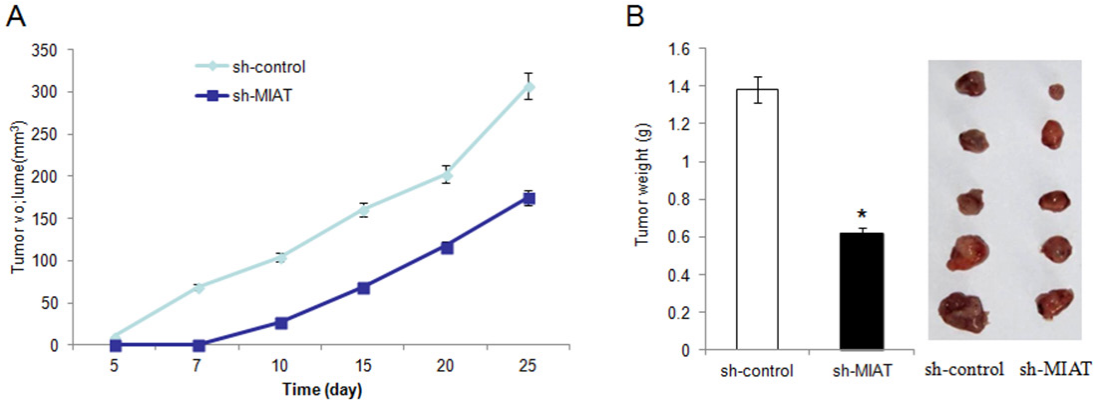
Knockdown of MIAT inhibited MDA-MB-231 cells growth *in vivo* **(A)**. Summary of tumor volume of nude mice which were measured in every 5 days in sh-control and sh-MIAT group, respectively. **(B)** Tumor weight and representation picture of tumor formation of xenograft in nude mice in sh-control and sh-MIAT group, respectively (each group n=5).

To verify our *in vitro* findings, we established an *in vivo* xenograft model in nude mice. Two lentivirus constructed cells, lv-sh-MIAT and lv-sh-control were well cultured and were injected subcutaneously. Tumors were allowed to grow for 4 weeks (n = 5 for each group). Tumor volumes were measured every 5 days. At the end of the fourth weeks, the mice were sacrificed and tumors excised. We found that MIAT knockdown significantly reduced the tumor volume of MDA-MB-231 cells compared with control group. And the tumor formation was later in MIAT knockdown group than the control group (Figure 5A and 5B). These data revealed that downregulation of MIAT inhibited breast cancer cell growth *in vivo*.

### MIAT functions as ceRNA to regulate DUSP7 expression by sponging miR-155-5p

**Figure 6:**
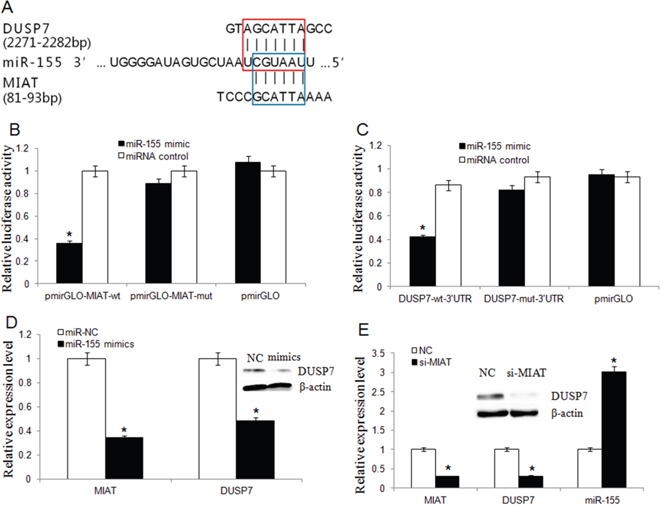
MIAT and DUSP7 shared a common miR-155-5p binding site **(A)** Bioinformatics analysis revealed that MIAT and DUSP7 shared a common miR-155-5p binding site. The red box represented the binding site for miR-155-5p on the DUSP7 mRNA 3′-UTR, and the blue box represented the binding site for miR-155-5p on MIAT. **(B)** Luciferase activity in HEK293T cells cotransfected with miR-155-5p mimics and luciferase reporters containing control vector, pmir-Glo-MIAT-wt and pmir-Glo-MIAT-mut (^*^P<0.05, Two-side Student's t-test; n = 3). miR-155-5p mimics reduced the luciferase activity of wt MIAT reporter vector but not that of mut MIAT reporter vector. **(C)** MiR-155-5p significantly inhibited the luciferase activity of the DUSP7 wt 3′-UTR but not that of the mutant (^*^P <0.05, Two-side Student's t-test; n = 3). **(D)** MiR-155-5p overexpression downregulated the endogenic MIAT and DUSP7 expression. (^*^P <0.05, Two-side Student's t-test; n = 3). Western blot assay showed that miR-155-5p overexpression silenced DUSP7 protein expression. **(E)** The miR-155-5p level was increased but DUSP7 mRNA level was decreased in MDA-MB-231 cells after MIAT knockdown. (Data are presented as mean ± SD, n=3. *P <0.05). Western blot assay showed that knockdown of MIAT triggered a significant silencing effect on endogenous DUSP7 protein.

We then analyzed whether the oncogenic activity of MIAT was through functions as ceRNA. A bioinformatics analysis showed that there were conserved binding sites for miR-155-5p on both MIAT and the 3′-UTR of DUSP7 mRNA (Figure 6A). MiR-155-5p can complementarily bind to the MIAT sequence between 81bp and 93bp, and its sequence is also complementary to the 3′-UTR sequence of DUSP7 mRNA between 2271bp and 2282bp.

To detect whether miR-155-5p was targeted and directly bound to MIAT, fragments of wild-type (wt) and mutated (mut) MIAT cDNA sequence containing the putative miR-155-5p recognition site was cloned. Dual reporter luciferase was performed in HEK293T cells. The results indicated that miR-155-5p mimics significantly decreased the luciferase activities of pmir-Glo-MIAT-wt but not pmir-Glo-MIAT-mut (Figure 6B). To explore that DUSP7 was a direct target of miR-155-5p, DUSP7 wt or mut 3′-UTR was subcloned into a luciferase reporter vector and co-transfected with miR-155-5p mimics or negative control into HEK293T cells. The results showed that miR-155-5p significantly inhibited the luciferase activity of the DUSP7- 3′-UTR but not that of the mutant (Figure 6C).

We transfected miR-155-5p mimics into MDA-MB-231 cell line and qRT-PCR analysis revealed that miR-155-5p overexpression could suppress both MIAT and DUSP7 mRNA and DUSP7 protein expression (Figure 6D). According to the ceRNA hypothesis, knockdown MIAT will result in freeing of miR-155-5p, and this miR-155-5p will target DUSP7 mRNA and trigger the downregulation of DUSP7. Thus, we detected whether downregulation of MIAT would influence miR-155-5p and DUSP7 expression. We transfected MIAT-siRNA into MDA-MB-231 cell to knockdown MIAT, then qRT-PCR and western blot revealed that the miR-155-5p was increased but DUSP7 mRNA and protein was decreased (Figure 6E).

**Figure 7:**
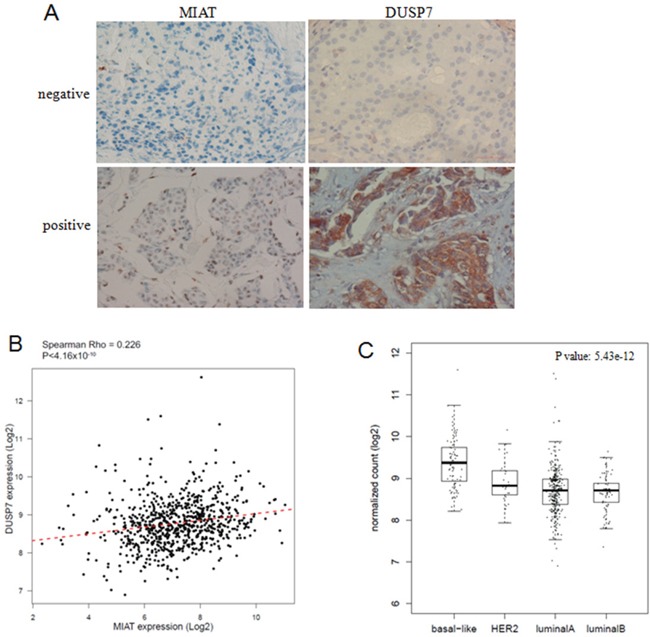
MIAT and DUSP7 had positive association **(A)** Consecutive sections of MIAT and DUSP7 expression in breast cancer TMA. MIAT RNA was detected by RNA Scope 2.0^®^ technology and DUSP7 protein was detected by IHC (×400). MIAT located at nucleus and DUSP7 located at cytoplasm. **(B)** The linear correlations between the expression level of MIAT and DUSP7 in breast cancer tissues from TCGA database (R=0.226, *P*<0.05). The data were obtained using the logistic regression analysis. **(C)** The DUSP7 expression was higher in basal-like breast cancer subtypes from TCGA database (*P*<0.05).

We detected the DUSP7 expression by IHC in TMA to analyze the association between MIAT and DUSP7. As shown in Figure 7A, the expression of MIAT and DUSP7 were all negative or all positive in the consecutive sections of breast cancer TMA. We found that there were six DUSP7 positive in eleven MIAT positive samples and two DUSP7 positive in nineteen MIAT negative samples. The result suggested the expression of MIAT and DUSP7 was positive association (Supplementary Table 4, *P*<0.05).

To validate our results, a total of 751 cases of breast cancer patients with MIAT and DUSP7 expression data from TCGA database were enrolled in this study and analyzed by Spearman correlation coefficient. As shown in Figure 7B, there were positive correlation between MIAT and DUSP7 in breast cancer patients (R=0.226, *P*<0.05). And we also found DUSP7 expression was higher in basal-like breast cancer (*P*<0.05) (Figure 6C). The results suggested that MIAT functions as oncogene partly through influencing DUSP7 expression in breast cancer through miR-155-5p.

## DISCUSSION

Emerging evidence indicated that LncRNAs play a significant role in cancer pathogenesis [21, 22]. However, the role of lncRNAs in breast cancer is still largely unknown. Our studies have revealed that the lncRNA MIAT is highly upregulated in breast cancer cell lines and breast cancer tissues.

It has been reported that MIAT knockdown significantly reduced the proliferation and mobility of endothelial cells and further involved in the pathogenesis of microvascular dysfunction [15]. LncRNA MIAT was specifically up-regulated in the plasma and aqueous humor of cataract patients. MIAT knockdown could affect the proliferation, apoptosis and migration of human lens epithelial cells (HLECs) upon oxidative stress [23]. In cancer research, lncRNA MIAT is selectively upregulated in neuroendocrine prostate cancer and might interact with Polycomb genes [9]. We found that all of the breast cancer cell lines showed higher MIAT level than normal breast cell line. And the highest expression level of MIAT was obtained in MDA-MB-231 cell line, may be due to the strong invasive and metastasis ability. Furthermore, knockdown of MIAT inhibited breast cancer cell proliferation, migration, invasion and EMT but promoted the rate of apoptosis. And downregulation of MIAT decreased tumor growth and delayed tumor formation *in vivo*. These data suggested that MIAT might promoted breast cancer malignant progression.

Moreover, MIAT expression level was positively associated with advanced TNM stage and lymphnode metastasis in breast cancer patients. Due to the limitation of clinical samples, further larger cohort study with follow-up information would provide reliable data to justify whether MIAT associated with prognosis or as a new biomarker for breast cancer.

To understand the mechanism of MIAT regulation in breast cancer, we indicated that MIAT shared miR-155-5p response element with DUSP7, which belongs to DUSP family. DUSPs are a heterogeneous group of protein phosphatases that can dephosphorylate both phosphotyrosine and phosphoserine/phosphothreonine residues within the one substrate. But the function of DUSP7 in cancer was not clear. Some reports suggested that DUSP7 exhibited reduced expression in particular cancers as tumour suppressors [24]. And the DUSP7 expression decreased if the ERK1/2 pathway was inhibited in microglia [25]. Our results indicted that DUSP7 was a direct target of miR-155-5p and its expression was higher in basal like breast cancer than other subtypes. There were positive correlation between DUSP7 expression and MIAT in breast cancer patients. We proposed that MIAT acted as a ceRNA to regulate DUSP7 mRNA by taking up miR-155-5p in breast cancer cells, which might be one of the mechanisms of MIAT as an oncogene.

In summary, our data suggested that MIAT may serve as an oncogene in breast cancer and promote breast cancer progression through interacting with miR-155-5p. Future studies will investigate the mechanisms of MIAT in breast cancer and the utility of MIAT as potential specific therapeutic target.

## MATERIALS AND METHODS

### TCGA dataset

To validate the potential role of MIAT and DUSP7 in breast cancer, we analyzed the Caner Genome Atlas project (TCGA dataset, http://cancergenome.nih.gov/) which provides more than 1000 breast cancer cases including gene expression data and follow-up information. Here, the lncRNA expression data were extracted from the level 3 RNASeqV2 data. A total of 751 cases of breast cancer patients with MIAT and DUSP7 expression data were enrolled in this study. In addition, we also collected 113 non-tumor breast tissues containing MIAT and DUSP7 expression data from TCGA data sets.

### Tissue samples

Breast tumor tissue microarrays (TMA) (HBre-Duc060CS) were obtained from Shanghai Outdo Biotech Co. (Shanghai, China). The array contains 30 cases of invasive ductal carcinomas and 30 normal breast tissues from the regions around cancers. None of the patients received adjuvant chemotherapy, immunotherapy, or radiotherapy before surgery. All experimental protocols were approved by The Human Research Ethics Committee from Harbin Medical University.

### Cells and culture conditions

Breast cancer cell lines and one normal breast cell line MCF-10A were obtained from the Shanghai Institutes for Biological Sciences, Chinese Academy of Sciences (Shanghai, China). MCF10A was cultured in F12/DMEM 1:1 medium. MCF-7 was cultured in MEM medium with sodium pyruvate 0.11 mg/ml and bovine insulin 0.01 mg/ml. T-47D and SK-BR-3 were cultured in DMEM medium. All cells were cultured with 10% fetal bovine serum (FBS), at 37°C and 5% CO_2_. MDA-MB-231, MDA-MB-468 and MDA-MB-453 cells were cultured in L-15 medium with 10% fetal bovine serum (FBS), at 37°C and no CO_2_.

### RNAscope^®^ 2.0 analysis for MIAT RNA detection

Hybridization was with target probes (probe symbols: a 20ZZ probe named Hs-MIAT targeting 330-1422 of NR_003491.3). The preamplifier, amplifier, label probe, and chromogenic detection procedures were according to the manufacturer's instructions (RNAscope^®^ 2.0 HD Reagent Kit, Advanced Cell Diagnostics, Hayward, CA, USA).

### Immunohistochemical staining

The tissue sections were dried at 60°C for 1 h. The tissue sections were dewaxed in xylene and rehydrated through graded alcohol concentrations using standard procedures. Antigen retrieval was performed in citrate buffer (pH 6.0) and autoclave at 121°C for 90 seconds. After washing in PBS (3 min ×3), sections were blocked with goat serum (Boster, Wuhan, China) in the room temperature for 30 min. Then each section was treated with DUSP7 rabbit polyclonal antibodies (bs-7928) (Bioss Antibodies, Inc.; at a dilution of 1:200 solution) at 4°C overnight. After washing in PBS (5 min ×3), each section was incubated with Polink-1 HRP DAB Detection System One-step polymer detection system for Rabbit antibody (ZSGB-BIO, Beijing, China) at room temperature for 20 min. After washing in PBS (3 min ×3), the slides were counterstained with hematoxylin. For negative controls, the primary antibody was substituted with PBS.

### Gene silencing by siRNA transfection and lentivirus-mediated transduction of shRNA

For the transfection of the miRNA mimics and siRNAs, MDA-MB-231 cells (2×10^5^) were seeded in 6-well plates. The following day, they were transfected with 100 nM of miRNA mimics or 50 nM siRNA using Lipofectamine 2000 Reagent (Life Technologies). The sequence of the miR-155-5p mimics was 5'-UUAAUGCUAAUCGUGAUAGGGGU-3' (sense) and 5'-CCCUAUCACGAUUAGCAUUAAUU-3' (antisense). The sequence of the MIAT siRNA was 5'-GGUGUUAAGACUUGGUUUCTT-3' (sense) and 5'-GAAACCAAGUCUUAACACCTT -3' (antisense). The sequences of the negative control siRNAs were 5'-UUCUCCGAACGUGUCACGUTT-3' (sense) and 5'-ACGUGACACGUUCGGAGAATT-3' (antisense). These sequences were synthesized by GenePharma Co., Ltd. (Shanghai, China). Lentivirus vector LV10 carrying MIAT siRNA sense sequence or control scramble siRNA sequence were purchased from GenePharma Co., Ltd. (Shanghai, China).

### RNA extraction and quantitative reverse transcription PCR

Total RNA was extracted using TRIzol Reagent (Life Technologies) according to the manufacturer's protocol. RNA was quantified and reverse transcribed into cDNA using the ReverTra Ace-α qPCR RT Kit (Toyobo, Japan). RT-PCR of the mature miRNAs was performed using miRcute miRNA First-Strand cDNA Synthesis Kit (Tiangen, Beijing, China). The qRT-PCR amplification protocol was conducted according to the user guide of the SYBR^®^ Green Realtime PCR Master Mix (Toyobo, Japan) on the ABI7500 Fast system. Melting curve analysis was used to monitor the specificity of the PCR products. GAPDH was used as a control. The MIAT primers were as follows: forward, 5'-GGACGTTCACAACCACACTG-3'; reverse, 5'-TCCCACTTTGGCATTCTAGG-3'; (Sangon Biotech, Shanghai, China). The DUSP7 primers were as follows: forward, 5'-CCAAGAAGTGTGGTGTCCTG-3'; reverse, 5'-ACAAAGTCGTAGGCGTCGTT-3'(Sangon Biotech, Shanghai, China). The GAPDH primers were as follows: forward, 5'-CGGAGTCAACGGATTTGGTCG-3'; reverse, 5' -TCTCGCTCCTGGAAGATGGTGAT-3'. The miR-155-5p primers were as follows: forward, 5'-GCTTCGGTTAATGCTAATCGTG-3'; reverse, 5'-CAGAGCAGGGTCCGAGGTA-3'. U6 was used as a control and the primers were as follows: forward, 5'-CTCGCTTCGGCAGCACA-3'; reverse, 5'-AACGCTTCACGAATTTGCGT-3'. All experiments were performed in triplicate. The qRT-PCR results were analyzed and expressed as relative miRNA or mRNA levels of the CT (cycle threshold) value, which was then converted to fold change.

### Luciferase report assay

The HEK293T cells were seeded at 1.5×10^4^/well in 96-well plates and co-transfected with 200 ng of pmri-GLO-MIAT-wt, pmri-GLO-MIAT-mut, pmri-GLO-DUSP7-wt, pmri-GLO-DUSP7-mut or pmri-GLO (Sangon biotech, China). 10 ng of pRL-TK (Promega, USA) were also co-transfected with miR-155-5p mimics or miRNA NC into HEK293T cells using Lipofectamine 2000 (Invitrogen, USA). The luciferase assay was performed by using the dual-luciferase reporter assay system (Promega) 48 h after transfection. Transfection was repeated in triplicate.

### Western blot

MDA-MB-231 cells transfected with LV10-sh-MIAT or LV10-sh-NC were harvested and lysed with cell lysis buffer for western blotting (Beyotime, Shanghai, China). The proteins (30μg per lane) were separated on 12% SDS-polyacrylamide gels and transferred into polyvinylidene fluoride (PVDF) membranes (Millipore, Billerica, MA, USA). Primary antibodies for rabbit-anti-DUSP7 (1:200) (bs-7928, Bioss Antibodies, Inc.), rabbit-anti-P21(1:500) (sc-397, Santa Cruz Biotechnology, Inc.), rabbit-anti-E-Cadherin (1:10000) (ab40772, Abcam, USA), rabbit-anti-Vimentin (1:2000); (ab92547, Abcam, USA) and rabbit-anti-β-actin (1:1000) (Bioss Antibodies, Inc.) were incubated at 4°C overnight. Binding of the primary antibody was detected using an enhanced chemiluminescence kit (ECL Amersham).

### Cell proliferation assays

MDA-MB-231 cells were seeded in 96-well plates and transfected with MIAT siRNA and negative control. After transfection for 4, 24, 48 and 72 hrs, 10μL of the CCK-8 reagent was added into each well, incubated in 37°C, 5% CO_2_ for 2 hrs, and cell growth was detected by an enzyme labeling instrument at 450 nm.

### Cell migration and invasion assays

MDA-MB-231 cells were transfected with MIAT siRNA or scramble siRNA, respectively. At 24 h after transfection, cells in serum-free media were placed into the upper chamber of an insert for migration assays (8-μm pore size, millipore) and invasion assays with matrigel (Sigma-Aldrich, USA). Media containing 10% FBS was added to the lower chamber. After 24 hours of incubation, the cells that had migrated or invaded through the membrane were stained with methanol and 0.1% crystal violet, imaged, and counted using an inverted microscope (Olympus, Tokyo, Japan). The amount of cells passing through the membrane from five different fields per sample at 200× selected in a random manner was used to determine the capacity of cell migration or invasion.

### Annexin V and PI

The Annexin V assay was used to detect cells in the early stage of apoptosis. PI staining was detected in the dead cells. The 2×10^5^ cells were stained for 15min in 100μl binding buffer with 5μl of FITC-labeled Annexin V and 5μl of propidium iodide (PI) at room temperature in the dark. Analysis was performed by flow cytometry within 1hr.

### The LIVE/DEAD^®^ viability/cytotoxicity assay

Calcein-AM and ethidium homodimer (EthD-1) double staining was used to discriminate live and dead cells. MDA-MB-231 cells were transfected with MIAT siRNA or scramble siRNA, and then removed growth media and wash cells gently with PBS. Prepared the combined LIVE/DEAD^®^ assay reagents according to the protocol, and add 150μl to each well of 96-well plate. Then put the plate in 37°C incubator for 20 min. Detected the plate with LB 942 TriStar^2^ of BERTHOLD. The living cells were observed using a 530 nm excitation filter, while the dead cells were observed using a 645 nm excitation filter. Calculated the percentage of live and dead cells according to the formula from the protocol.

### Immunofluoresence assay

MDA-MB-231 cells transfected with LV10-sh-MIAT or LV10-sh-NC were fixed with 4% polyformaldehyde for 10 min at room temperature. Cells were washed with PBS for three times and incubated with 0.2% Triton X100 for 10 min at room temperature. Nonspecific binding sites were blocked with 5% goat serum albumin for 30 min. These cells were incubated with the primary antibody (E-Cadherin, 1:200 and Vimentin, 1:200; ab40772 and ab92547, Abcam, USA) overnight at 4°C, and then incubated with the secondary antibody conjugated with FITC (Bioss Antibodies, Inc.) for 1 hr at room temperature, followed by incubation with DAPI (4′, 6-Diamidino-2-phenylindole dihydrochloride) (ab104139, Abcam, USA) for 5 min. These cells were subsequently observed using the Olympus microscope.

### Xenograft mouse model

MDA-MB-231 cells (8×10^6^) stably expressing control shRNA or shRNA-MIAT were subcutaneously injected into either side of flank area of 4-week-old female athymic nude mice (n=5 mice per group). Tumor volumes were measured (0.5×length×width^2^) in mice on a weekly basis. After 4 weeks, the nude mice were sacrificed and the tumor tissues were excised and fixed in 4% paraformaldehyde solution for further study. All animal experiments were performed in the animal laboratory center of the Second Affiliated Hospital of Harbin Medical University and in accordance with the Guide for the Care and Use of Laboratory Animals published by the US National Institutes of Health (NIH publication number 85–23, revised 1996). The protocol was approved by the Animal Care and Use committee of the Second Affiliated Hospital of Harbin Medical University.

### Statistical analysis

All data was presented as means±SEM. All experiments were repeated at least three times. Comparison of two experimental groups was evaluated by the unpaired Student's t-test. All statistical analyses were performed by using the SPSS software version 17.0. The chi-square test was used to compare MIAT expression between breast cancer tissues and paired normal breast tissues and the association between MIAT expression and clinicopathologic parameters. The correlation between MIAT and DUSP7 expression was computed by Spearman coefficient. *P*<0.05 was considered to be statistically significant.

## SUPPLEMENTARY MATERIALS AND TABLES


