# Supplementary material for: Long non-coding RNA MIAT promotes breast cancer progression and functions as ceRNA to regulate DUSP7 expression by sponging miR-155-5p
Source: Oncotarget. 2017 Jul 12;8(44):76153–64. doi: 10.18632/oncotarget.19190 (PMC5652694; doi:10.18632/oncotarget.19190)
Supplement: Supplementary file 1 [file oncotarget-08-76153-s001.pdf]

Long non-coding RNA MIAT promotes breast cancer progression and functions as ceRNA to regulate DUSP7 expression by sponging miR-155-5p

SUPPLEMENTARY MATERIALS

Supplementary Table 1: The expression of MIAT in paired breast cancer tissues

	MIAT		χ^2	*P*
	-	+		
normal tissues	28	2	7.95	0.0048
cancer tissues	19	11		

Supplementary Table 2: Association between MIAT level with TNM stage of breast cancer patients

TNM stage	-	+	*P*
I, II	19	1	<.0001
III	0	10	

Supplementary Table 3: Association between MIAT level with Lymphnode metastasis of breast cancer patients

MIAT	LN	
	-	+
-	12	7
+	0	11

Chi-square test, *P*=0.0007.

Supplementary Table 4: The expression of DUSP7 in MIAT positive and negative breast cancer tissues

MIAT	DUSP7	
	-	+
-	17	2
+	5	6

Chi-square test, *P*=0.0086.
